# Semi-automated validation and quantification of CTLA-4 in 90 different tumor entities using multiple antibodies and artificial intelligence

**DOI:** 10.1038/s41374-022-00728-4

**Published:** 2022-01-29

**Authors:** David Dum, Tjark L. C. Henke, Tim Mandelkow, Cheng Yang, Elena Bady, Jonas B. Raedler, Ronald Simon, Guido Sauter, Maximilian Lennartz, Franziska Büscheck, Andreas M. Luebke, Anne Menz, Andrea Hinsch, Doris Höflmayer, Sören Weidemann, Christoph Fraune, Katharina Möller, Patrick Lebok, Ria Uhlig, Christian Bernreuther, Frank Jacobsen, Till S. Clauditz, Waldemar Wilczak, Sarah Minner, Eike Burandt, Stefan Steurer, Niclas C. Blessin

**Affiliations:** 1 Institute of Pathology, University Medical Center Hamburg-Eppendorf, Hamburg, Germany; 2 College of Arts and Sciences, Boston University, Boston, MA USA

**Keywords:** Molecular imaging, Immunochemistry, Super-resolution microscopy, Tumour biomarkers, Bioinformatics

## Abstract

CTLA-4 is an inhibitory immune checkpoint receptor and a negative regulator of anti-tumor T-cell function. This study is aimed for a comparative analysis of CTLA-4^+^ cells between different tumor entities. To quantify CTLA-4^+^ cells, 4582 tumor samples from 90 different tumor entities as well as 608 samples of 76 different normal tissue types were analyzed by immunohistochemistry in a tissue microarray format. Two different antibody clones (MSVA-152R and CAL49) were validated and quantified using a deep learning framework for automated exclusion of unspecific immunostaining. Comparing both CTLA-4 antibodies revealed a clone dependent unspecific staining pattern in adrenal cortical adenoma (63%) for MSVA-152R and in pheochromocytoma (67%) as well as hepatocellular carcinoma (36%) for CAL49. After automated exclusion of non-specific staining reaction (3.6%), a strong correlation was observed for the densities of CTLA-4^+^ lymphocytes obtained by both antibodies (*r* = 0.87; *p* < 0.0001). A high CTLA-4^+^ cell density was linked to low pT category (*p* < 0.0001), absent lymph node metastases (*p* = 0.0354), and PD-L1 expression in tumor cells or inflammatory cells (*p* < 0.0001 each). A high CTLA-4/CD3-ratio was linked to absent lymph node metastases (*p* = 0.0295) and to PD-L1 positivity on immune cells (*p* = 0.0026). Marked differences exist in the number of CTLA-4^+^ lymphocytes between tumors. Analyzing two independent antibodies by a deep learning framework can facilitate automated quantification of immunohistochemically analyzed target proteins such as CTLA-4.

## Abstract (web summary)

A convolutional neural network (U-Net) for the assessment of aberrant CTLA-4 antibody staining using two independent antibody clones (MSVA-152R and CAL49) was trained and validated on 4582 tumor samples in this study. The deep learning-based framework facilitated automated CTLA-4 quantification in more than 90 different tumor entities via compensating for individual antibody shortcomings.

## Introduction

CTLA-4 (cytotoxic T-lymphocyte-associated protein 4, CD152) is an important inhibitory immune checkpoint receptor. It is expressed on various subtypes of T-lymphocytes including CD4^+^ and CD8^+^ T-cells as well as regulatory T-cells^1^. CTLA-4 can compete with its stimulating counterpart CD28 for ligand binding to CD80 and CD86^2,3^. CD28 co-stimulation is required for T-cell activation, whereas CTLA-4 inhibits T-cell response by opposing the actions of CD28-mediated co-stimulation^2,3^. Even though CTLA-4 is also expressed on activated CD8^+^ cytotoxic T-cells, the major physiologic role of CTLA-4 appears to be through down-modulation of non-regulatory T-cell activity and supportively enhancement of regulatory T-cell suppressive activity^1,4–6^. The CTLA-4 pathway is a commonly targeted pathway in cancer immunotherapy. For example, the CTLA-4 inhibitor Ipilimumab alone or in combined therapy has been approved for the treatment of advanced malignant melanoma, renal cell and microsatellite instability-high colorectal cancer by the Food and Drug Administration (FDA)^7^.

 Given the pivotal role of CTLA-4 as a successfully used drug target, the prevalence and topographic distribution of CTLA-4^+^ lymphocytes and lymphocyte subclasses is of interest. Most studies analyzing CTLA-4 in cancer have employed flow cytometry or RNA based methods^1,8^. Because these techniques are best applicable to unfixed tissues which is unavailable from most tumors in routine praxis, studies on CTLA-4 in cancer mostly involved limited numbers of samples from frequently occurring tumor entities such as malignant melanoma (*n* = 56–470)^8,9^, breast (*n* = 928–1217)^10^, colorectal (*n* = 439–1003)^10–12^ and renal cell cancers (*n* = 813–928)^10,12,13^. Studies on less common tumor entities and larger patient cohorts require the use of routinely processed formalin fixed tissues but were so far hindered by a relative lack of CTLA-4 antibodies suitable for immunohistochemistry (IHC). Antibodies with documented specificity on unprocessed native target protein often show disappointing results on formalin fixed tissues^14–16^. Potential shortcomings include a lack of target protein staining, an unfavorable signal-to-noise ratio resulting in non-specific background staining, and antibody cross-reactivity resulting in a distinct staining of structures not containing the target protein^14,15^.

In order to determine the prevalence of CTLA-4^+^ lymphocytes in a broad range of different tumor entities, a set of preexisting tissue microarrays (TMAs) was analyzed that included >4000 tumor samples from 90 types and subtypes as well as 76 different normal tissue categories. To compensate for possible shortcomings of CTLA-4 immunohistochemistry, two different CTLA-4 antibodies were used in combination with an artificial intelligence approach for automated discrimination of true from aberrant antibody staining.

## Materials and methods

### Tissue microarrays (TMAs)

Our normal tissue TMA was composed of 8 samples from 8 different donors for each of 76 different normal tissue types (608 samples on one slide). The cancer TMAs contained a total of 5706 primary tumors from 134 tumor types and subtypes. Detailed histopathological data such as grade, pT or pN information were available for >2600 cancers (Table 2). Data on the PD-L1 status of tumor-/inflammatory cells^17^ and the density of CD3^+^ T-cells^17^ were obtained in a previous study. The composition of normal and cancer TMAs is described in the results section. All samples were selected from the archives of the Institutes of Pathology, University Hospital of Hamburg, Germany, the Institute of Pathology, Clinical Center Osnabrueck, Germany, and Department of Pathology, Academic Hospital Fuerth, Germany. Tissues were fixed in 4% buffered formalin and then embedded in paraffin. The TMA manufacturing process was described earlier in detail^18,19^. In brief, one tissue spot (diameter: 0.6 mm) was transmitted from a cancer containing donor block to an empty recipient paraffin block. The use of archived remnants of diagnostic tissues for TMA manufacturing, their analysis for research purposes, and patient data were according to local laws (HmbKHG, §12) and analysis had been approved by the local ethics committee (Ethics commission Hamburg, WF-049/09). All work has been carried out in compliance with the Helsinki Declaration.

### Immunohistochemistry (IHC)

Freshly cut 4-µm TMA sections were immunostained on one day and in one experiment. Slides were deparaffinized and exposed to heat-induced antigen retrieval for 5 min in an autoclave at 121 °C in a pH 7.8 buffer. Primary antibody specific for CTLA-4 (rabbit recombinant, clone MSVA-152R, Cat#: 3451–152 R, MS Validated Antibodies GmbH, Hamburg, Germany, 1:50 and rabbit recombinant, clone CAL49, Cat#: ab237712, Abcam, Cambridge, USA, 1:100) were applied at 37 °C for 60 min. Bound antibody was then visualized using the EnVision Kit (Agilent DAKO, Santa Clara, USA) according to the manufacturer’s directions.

**Fig. 1:**
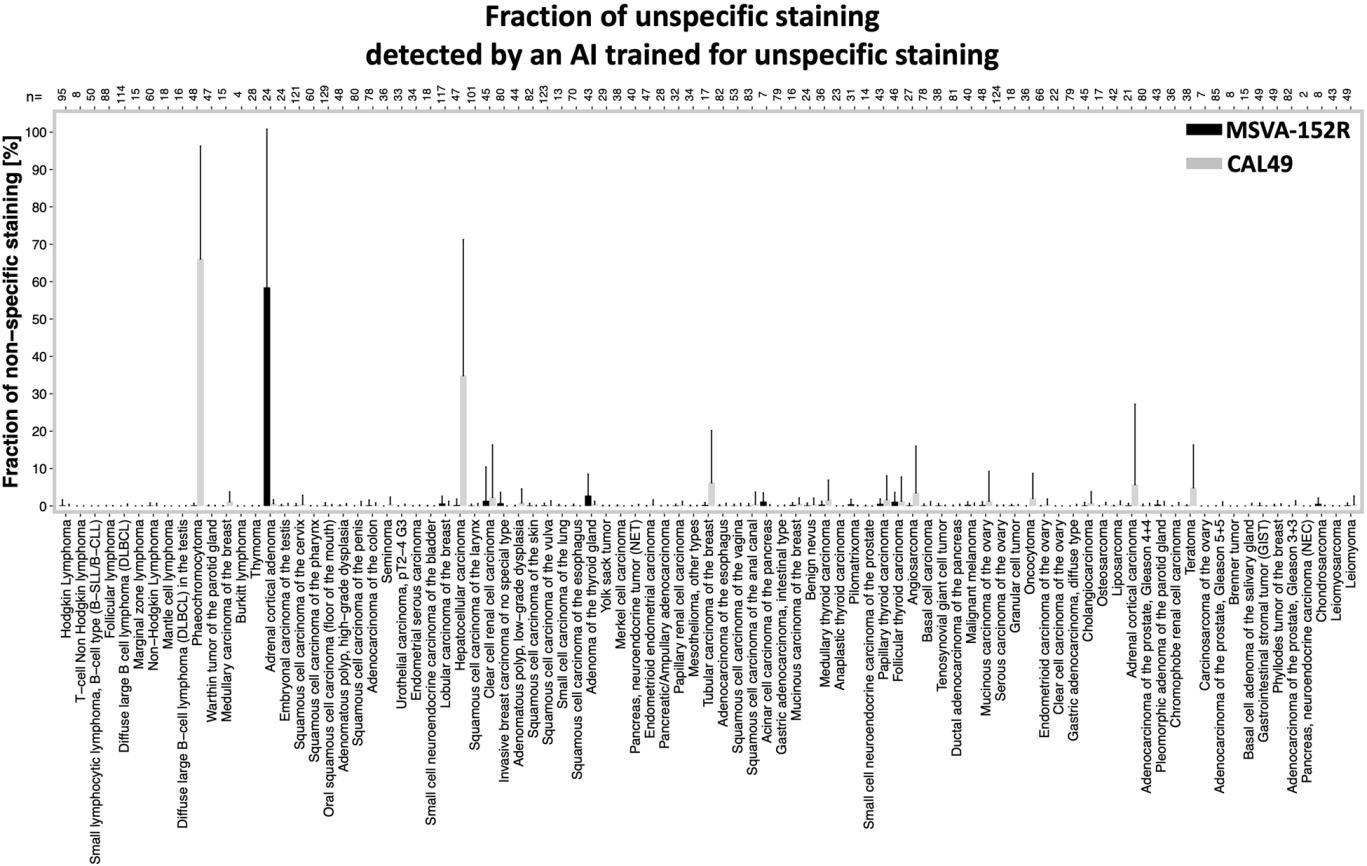
Fraction of non-specific staining detect by an AI framework trained for non-specific staining. The mean fraction of non-specific stained cells is shown for both CTLA-4 antibody clones MSVA-152R (black) and CAL49 (grey). Error bars indicate standard deviations.

For multiplex fluorescence IHC a freshly cut 4-µm healthy human tonsil was used. The experimental procedure was performed according to the manufacturer’s instructions (AKOYA). Slides were initially boiled in an autoclave (30 min at 100–120 °C in pH9 buffer) for antigen retrieval. The antibody panel consisted of a CD3 antibody for T-cell detection (rabbit polyclonal, Cat#: IR503, Agilent DAKO, Santa Clara, USA, undiluted), MSVA-152R, and CAL49 for CTLA-4 detection. The OPAL dye kit (Cat# NEL811001KT, AKOYA Biosciences, Menlo Park, California, United States) was used to detect the primary antibodies CD3 (OPAL 520), MSVA-152R (OPAL 570), and CAL49 (OPAL 690). These were combined with diamidino-2-phenylindole (DAPI) staining. One cycle of antibody staining included peroxidase blocking, application of the primary antibody, detection with a secondary HRP-conjugated antibody, fluorescence dye detection, and removal of the bound antibodies by microwave treatment (5 min at 100 °C and 5 min at a mean temperature of 93 °C). This cycle was repeated two times for the remaining antibodies. Slides were subsequently counterstained with DAPI and mounted in an antifade solution. To measure the co-expression of both CTLA-4 antibody clones in human tonsil (Fig. S1A, B) the CTLA-4 density and expression level were analyzed: Correlation analysis of the CTLA-4 expression level confirmed a high degree of co-expression (*r* = 0.81, *p* < 0.0001; Fig. 1C). In addition, the density of CTLA-4^+^ cells of both clones was highly concordant in 35 representative areas (*r* = 0.85, *p* < 0.0001, Fig. S1C).

### Deep learning-based image analysis

The slides were scanned using Leica’s Aperio AT2 slide scanner. The digital images were analyzed using a two-stage approach combining a convolutional neural network (U-Net) for automated quantification of CTLA-4^+^ cells (1) and a deep neural network (DeepLab3^+^) for the detection of non-specific (2) CTLA-4 staining (Fig. S2).

1. The U-Net deep learning system for cell identification was trained and validated as described earlier^20^. In brief, thresholding was used to label cell nuclei and the background of the first 500 patients. After manual correction of this training set the U-Net was trained for 300,000 iterations (∼30 epochs). The trained U-Net was used to analyze/label further 500 patients, which were also manually corrected. A new U-Net was trained based on these two training sets to label the next 500 patients. The process was used to continuously increased the training set until 3306 (75%) TMA spots (from 90 different tumor entities), were successfully labeled, manually corrected, and used for the training of the final U-Net for cell segmentation. Of note, to avoid introducing potential bias by selective manually correction two trained pathologist were relabeling and manually correcting the labels. The threshold for CTLA-4 positivity was visually investigated. The area in square millimeter of each spots was calculated by a pretrained U-Net algorithm^21^.
2. The DeepLab3^+^ deep learning system for detecting aberrant antibody staining was trained on 75% of cases for every tumor entity to assure a balanced training input. A pathologist identified regions and TMA cores showing non-specific staining so that thresholding could be used to label regions of non-specific staining as well as background. Comparison of the staining pattern from both CTLA-4 clones for the same consecutive TMA spot enabled the identification of false positive antibody staining. Specific CTLA-4 staining was labeled as background. The mean fraction of non-specific stained cells per tumor entity is shown in Fig. 1. Tumor samples with 5% or more cells with non-specific staining were identified as a case driven by false positive staining and excluded from further analysis (Fig. S3). Thus, the mean CTLA-4 density (cells/mm^2^) of both antibodies was based on TMA cores showing 4% or less non-specific CTLA-4 staining. The performance of both deep learning systems was evaluated by calculating the area under (AUC) receiver operating characteristics (ROC) using the remaining (25%) of patients as a validation set (Fig. S3). Python version 3.8^22^ and the Visiopharm software package (Hoersholm, Denmark) were used to label, train, and validate the deep learning systems.

### Statistical analysis

Statistical calculations were performed with R version 3.6.1 (The R foundation)^23,24^ and JMP Pro 15 software package (SAS Institute Inc., NC, USA)^25^. Contingency tables and the Chi-square test were used to search for associations between the density of CTLA-4 and tumor phenotype. All *p* values were two-sided, and *p* < 0.05 were considered as significant.

## Results

### CTLA-4 in normal tissues

**Fig. 2:**
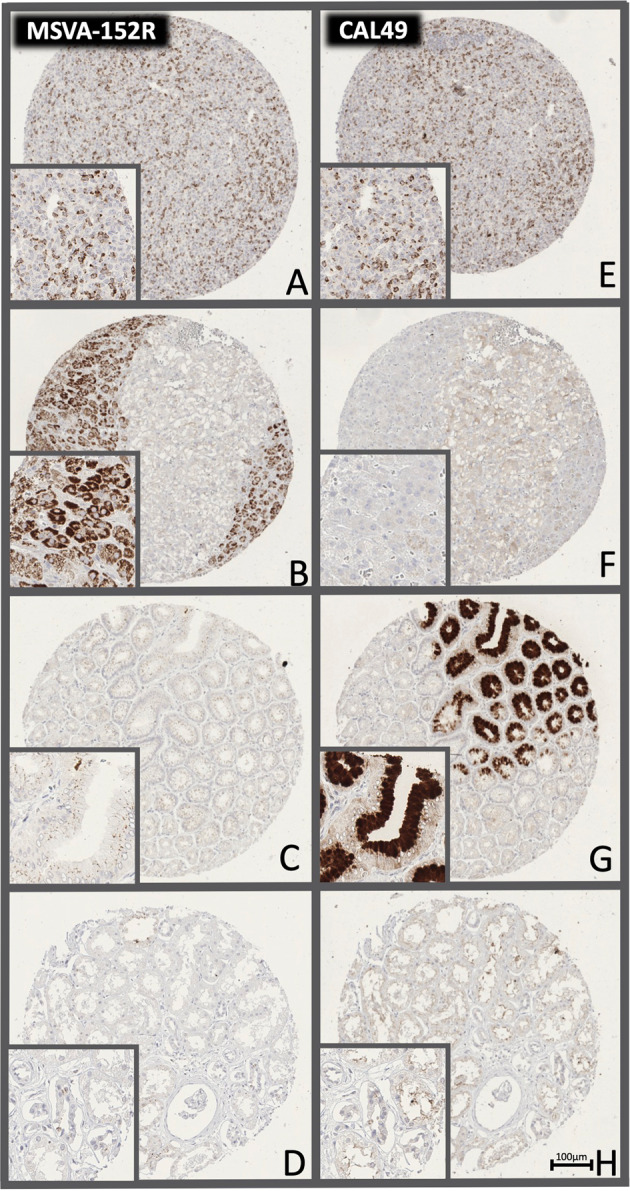
CTLA-4 immunostaining of normal tissues. The panels show for the antibody MSVA-152R a strong membranous positivity of a subset of lymphocytes in the tonsil (**A**), a strong cytoplasmatic staining of the adrenal cortex (**B**) and a cytoplasmic granular staining in a fraction of superficial epithelial cells of the stomach (**C**) and of renal tubuli (**D**). For the antibody CAL49, a strong membranous positivity of the same subset of lymphocytes in the tonsil (**E**), a weak cytoplasmatic staining of the adrenal medulla (**F**), a strong cytoplasmic staining superficial epithelial cells of the stomach (**G**), and an apical membranous staining of renal tubuli (**H**) is seen. The images (**A**–**D**) and (**E**–**H**) are from consecutive tissue sections and taken at 20x magnification.

Using both antibodies, a strong and distinct, predominantly membranous CTLA-4 immunostaining was seen in a subset of T-lymphocytes. Both antibodies also stained thyroidal colloid. In addition, for MSVA-152R, an intense granular cytoplasmic staining could be seen in adrenocortical cells and decidua cells while a less conspicuous granular staining could be observed in the apical cytoplasm of tall columnar cells of the epididymis, pancreatic acinar cells, hepatocytes, and gastrointestinal surface epithelium cells. For CAL49 a strong cytoplasmic staining was seen in gastric surface epithelial cells and sebaceous glands while a weak cytoplasmic staining was seen in medullary cells of the thyroid and a weak to moderate staining of apical membranes in selected renal tubuli. All these stainings which were distinct when applying one antibody but absent for the other antibody were considered antibody-specific cross-reactivities. Although thyroidal colloid was stained by both antibodies, this staining was also rather considered cross-reactive because the function of CTLA-4 is not consistent with a role as a thyroidal colloid component. Representative images are shown in Fig. 2.

### CTLA-4 antibody validation in tumor tissues

**Fig. 3:**
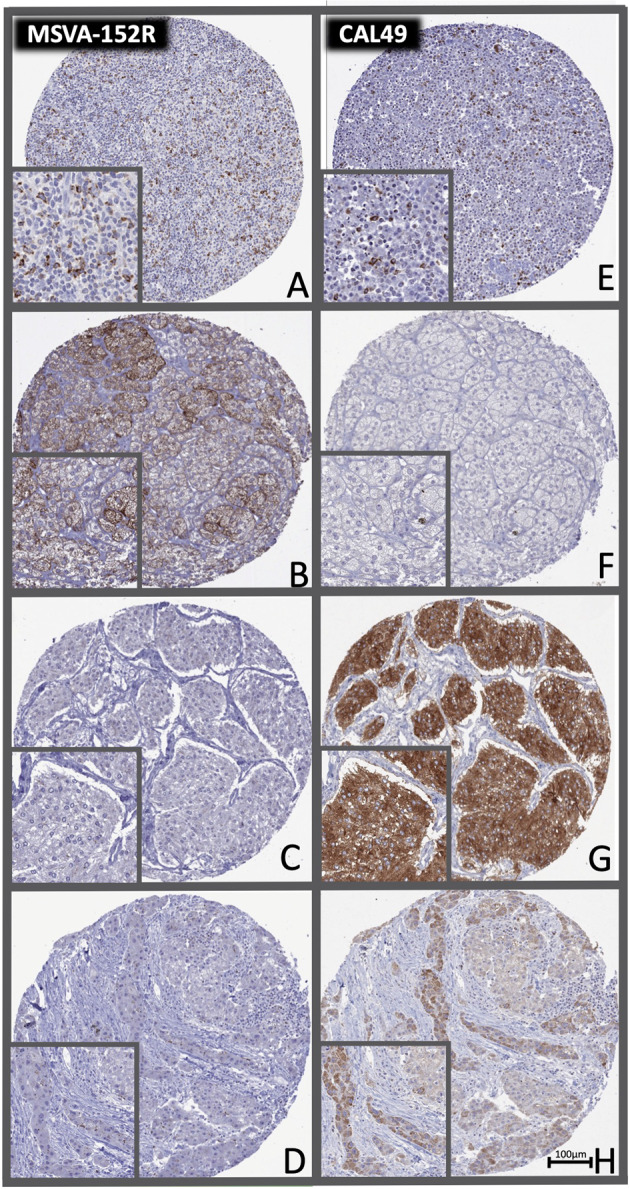
Distinct target staining and non-overlapping cross-reactivities of two CTLA-4 antibodies. The panels show for the antibody MSVA-152R a strong staining of a subset of lymphocytes in a Hodgkin’s lymphoma (**A**), a strong cytoplasmatic staining of an adrenocortical adenoma (**B**) absence of staining in a pheochromocytoma (**C**), and a staining of few lymphocytes in a hepatocellular carcinoma (**D**). For the antibody CAL49, an equally strong staining of the identical subset of lymphocytes in a Hodgkin’s lymphoma is seen (**E**), while staining is lacking in an adrenocortical adenoma (**F**), and a cytoplasmic staining occurs in a pheochromocytoma (**G**) and a hepatocellular carcinoma (**H**). The images (**A**–**D**) and (**E**–**H**) are from consecutive tissue sections and taken at 20x magnification.

A total of 9405 images from 90 different tumor entities were used to train and validate a deep learning-based approach for detecting non-specific staining (Fig. S2). Our approach identified a high fraction of non-specific staining for MSVA-152R in adrenal cortical adenoma (58%) and for CAL49 in pheochromocytoma (66%) as well as hepatocellular carcinoma (35%, Fig. 1). Non-specific staining for both antibodies was found in 1% to 8% of cells in malignant melanomas, adrenocortical carcinomas, renal and thyroidal tumors. Representative tumor images are shown in Fig. 3. After automated exclusion of perceived non-specific staining reaction in 126 cases (2.7%) of the 4723 cases stained with MSVA-152R and in 213 (4.5%) of the 4682 cases stained with CAL49, a strong correlation was observed for the densities of CTLA-4^+^ cells obtained by our two antibodies (*r* = 0.93; *p* < 0.0001; Fig. S4). For all further analyses, the average densities of CTLA-4^+^ cells obtained by both antibodies were used for each patient except for tumor samples with >5% of non-specific staining. In these cases, only the data from the antibody with specific staining was utilized.

### CTLA-4 in tumor tissues

**Fig. 4:**
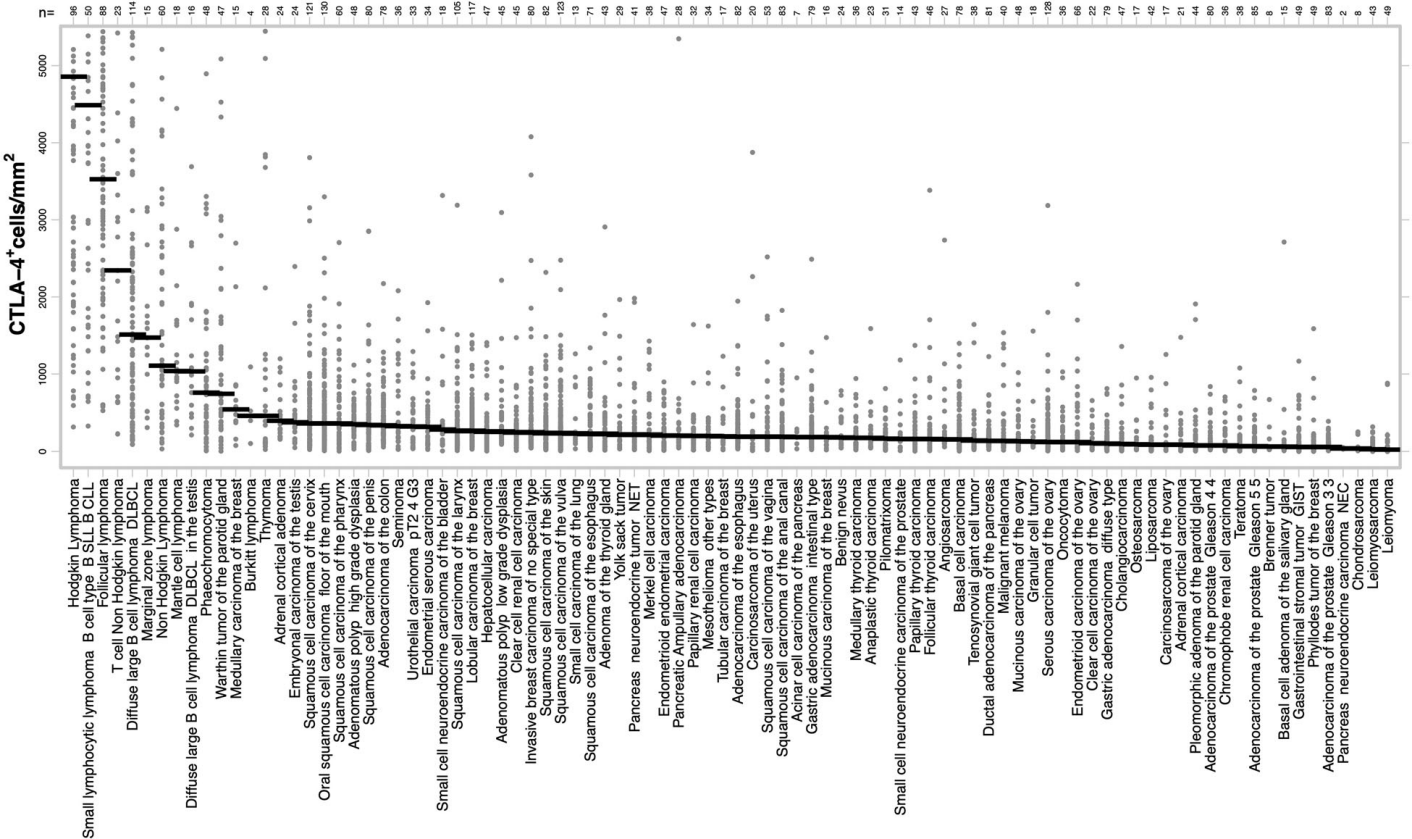
CTLA-4 density in human neoplasms. Distribution of the CTLA-4^+^ cell density (cell/mm^2^) across 90 different human tumor entities. In total 4582 tumor samples, represented by gray dots, were analyzed. The vertical bars indicate the mean density per entity.

A total of 4582 different patients from 90 different tumor entities—after exclusion of 339 (3.6%) of 9405 cases—were analyzed in this study. The mean density of CTLA-4^+^ cells was 674 ± 1482 cells/mm^2^ and ranged from 71 ± 175 cells/mm^2^ in leiomyoma to 5916 ± 3826 cells/mm^2^ in Hodgkin’s lymphoma (Fig. 4; Table S1). A comparison of the densities of CTLA-4^+^ cells in different tumor categories identified highest values in lymphomas (3642 ± 3207 cells/mm^2^), biphasic (609 ± 1057 cells/mm^2^) as well as germ cell tumors (401 ± 468 cells/mm^2^) and the lowest in mesothelial (281 ± 319 cells/mm^2^) as well as mesenchymal neoplasms (145 ± 268 cells/mm^2^; Table 1). Within epithelial tumors, the density of CTLA-4^+^ cells was higher in squamous cell (421 ± 469 cells/mm^2^) and urothelial carcinomas (418 ± 347 cells/mm^2^) than in adenocarcinomas (268 ± 375 cells/mm^2^) and renal cell neoplasms (256 ± 269 cells/mm^2^; Table 1). A comparison with histologic parameters and PD-L1 status revealed significantly higher rates of CTLA-4^+^ cells in tumors with low pT category (*p* < 0.0001), absent lymph node metastases (*p* = 0.0031), and PD-L1 expression in tumor cells or inflammatory cells (*p* < 0.0001 each; Table 2). Similar associations were also seen within the more homogeneous subgroups of adenocarcinomas and squamous cell carcinomas (data not shown). Across 908 squamous cell carcinomas, a high density of CTLA-4^+^ cells was linked to a positive HPV status (*p* = 0.0130; Table 2). Unequivocal CTLA-4 immunostaining of tumor cells was not seen in our patients.

**Table 1:** CTLA-4^+^ cell densities (cells/mm2) and CTLA-4/CD3-ratio in different tumor categories.

Characteristic	Patient number (%)	Mean density of both CTLA4-Ab	*p* value	Patient number (%)	CTLA4/CD3-ratio	*p* value
Total	4582	673 (±1482) median: 214		4292	17 (±52) median: 2	
Benign/malignant			<0.0001			0.3442
Malignant	3470 (75.7%)	734 (±1621)		3259 (75.9%)	15 (±40)	
Benign	432 (9.4%)	395 (±663)		452 (10.5%)	18 (±65)	
Origin			<0.0001			<0.0001
Lymphoma	424 (9.3%)	3642 (±3207)		297 (6.9%)	5 (±7)	
Biphasic	133 (2.9%)	609 (±1057)		128 (3.0%)	11 (±29)	
Germ cell tumor	127 (2.8%)	401 (±468)		144 (3.4%)	8 (±24)	
Epithelial	2954 (64.5%)	335 (±471)		2855 (66.5%)	18 (±48)	
Melanocytic	64 (1.4%)	307 (±340)		70 (1.6%)	25 (±43)	
Mesothelial	34 (0.7%)	281 (±320)		35 (0.8%)	15 (±31)	
Mesenchymal	235 (5.1%)	145 (±268)		255 (5.9%)	9 (±31)	
Lymphoma			<0.0001			0.0873
Hodgkin’s lymphoma	96 (2.1%)	5916 (±3826)		50 (1.2%)	3 (±2)	
NHL B-cell	305 (6.7%)	2997 (±2710)		236 (5.5%)	5 (±8)	
NHL T-cell	23 (0.5%)	2701 (±1949)		11 (0.3%)	6 (±7)	
Epithelial tumors			<0.0001			0.2193
Squamous	908 (19.8%)	421 (±469)		861 (20.1%)	20 (±45)	
Urothelial	33 (0.7%)	418 (±347)		37 (0.8%)	15 (±46)	
Adeno	1477 (32.2%)	268 (±375)		1419 (33.1%)	16 (±43)	
Renal	113 (2.5%)	256 (±269)		120 (2.8%)	17 (±34)	
Adenocarcinomas			<0.0001			0.1630
Lower GI	89 (1.9%)	448 (±343)		78 (1.8%)	20 (±33)	
Breast	245 (5.4%)	411 (±505)		220 (5.1%)	18 (±52)	
Thyroid gland	248 (5.4%)	300 (±452)		93 (2.2%)	23 (±49)	
Hep/Biliary/Pancreas	211 (4.6%)	258 (±432)		207 (4.8%)	17 (±45)	
Gyn	345 (7.5%)	256 (±336)		332 (7.7%)	15 (±40)	
Upper GI	240 (5.2%)	249 (±295)		221 (5.1%)	17 (±54)	
Adrenal cortical	21 (0.5%)	221 (±324)		25 (0.6%)	14 (±28)	
Prostate	78 (1.7%)	110 (±124)		242 (5.6%)	8 (±23)

**Table 2:** Association between the CTLA-4^+^ cell density (cells/mm2) as well as the CTLA4/CD3-ratio and clinicopathological parameters.

Characteristic	Patient number (%)	CTLA4^+^ cell density	*p* value	Patient number (%)	CTLA4/CD3-ratio	*p* value
Total	4582	673 (±1482) median: 214		4292	17 (±52) median: 2	
Pathological tumor stage			<0.0001			0.1846
pT1	763 (16.7%)	410 (±570)		740 (17.2%)	20 (±56)	
pT2	746 (16.3%)	350 (±467)		714 (16.6%)	18 (±46)	
pT3	839 (18.3%)	273 (±364)		710 (16.5%)	16 (±39)	
pT4	341 (7.4%)	306 (±345)		328 (7.6%)	15 (±30)	
Missing data	1893 (41.3%)	-		1800 (41.9%)	-	
Pathological nodal stage			0.0031			0.0354
pN−	839 (18.3%)	373 (±491)		794 (18.5%)	21 (±55)	
pN+	1003 (21.9%)	312 (±398)		962 (22.4%)	16 (±37)	
Missing data	2740 (59.8%)	-		2536 (59.1%)	-	
PD-L1 on tumor cells			<0.0001			0.0026
Negative	2583 (56.4%)	628 (±1382)		2498 (58.2%)	16 (±49)	
Positive	662 (14.4%)	920 (±1744)		605 (14.1%)	23 (±64)	
Missing data	1337 (29.2%)	-		1189 (27.7%)	-	
PD-L1 on immune cells			<0.0001			0.1010
Negative	2063 (45.0%)	310 (±534)		2068 (48.2%)	19 (±59)	
Positive	1371 (29.9%)	1233 (±2059)		1231 (28.7%)	16 (±45)	
Missing data	1148 (25.1%)	-		993 (23.1%)	-	
HPV			0.0130			0.9020
Negative	326 (7.1%)	393 (±392)		291 (6.8%)	25 (±56)	
Positive	243 (5.3%)	489 (±524)		221 (5.2%)	25 (±40)	
Missing data	4013 (87.6%)	-		3780 (88.0%)	-	

### CTLA-4/CD3 in tumor tissues

An elevated CTLA-4 density was linked to a high CD3^+^ T-cell density (*r* = 0.69, *p* < 0.0001, Fig. S5). If the ratio of the CTLA-4^+^ cell density and the CD3^+^ T-cell density was used as an analyte, most associations seen for the CTLA-4 density were no longer found. There was, however, a significant association between a high CTLA-4/CD3-ratio and absence of nodal metastases in 1756 cancer samples (*p* = 0.0354, Table 2). A high CTLA-4/CD3-ratio was linked to PD-L1 positivity on immune cells (*p* = 0.0026, Table 2). The CTLA-4/CD3-ratio also showed differences between different tumor categories: Lowest values were found in lymphomas (5 ± 7) and germ cell tumors (8 ± 24) while highest values were seen in melanocytic (25 ± 43) as well as epithelial tumors (18 ± 48, *p* < 0.0001, Table 1). Even though, the CTLA-4 density was highly variable in epithelial tumors (ranging from 256 to 421 cells/mm^2^; *p* < 0.0001) the CTLA-4/CD3-ratio was similar in different origins of epithelial tumors (ranging from 15 to 20; *p* = 0.2193; Table 2).

## Discussion

The data from this study demonstrate the feasibility of a reliable and precise high-throughput quantification of lymphocyte subpopulations by employing an AI supported multiple antibody approach.

Two different CTLA-4 antibodies were used for this study because the use of multiple independent antibodies is the only practically feasible approach for validating lymphocyte marker antibodies for immunohistochemistry on formalin fixed tissues. Although the International Working Group for Antibody Validation (IWGAV) has proposed that antibody validation for immunohistochemistry could alternatively include a comparison of the IHC findings with expression data obtained by another independent method^26^, this approach is not practical for immune cell markers due to the widespread distribution of immune cells across virtually all tissues. That both applied antibodies identified almost identical subsets of lymphocytes in multicolor analyses demonstrates, provides strong evidence for both antibodies recognizing CTLA-4 in formalin fixed tissues. The comprehensive screening of 76 different normal tissue categories also indeed identified multiple tissue structures that were significantly stained by one antibody but not by the other. While a staining of the target protein can be expected to occur with every suitable antibody it is likely that cross-reactivities are more antibody specific and therefore will involve non-overlapping tissues and cell types. The CAL49 staining observed in stomach and kidney epithelium as well as the MSVA-152R staining in adrenal gland, decidua cells and other epithelial cells are thus considered antibody cross-reactivities. Cross-reactivities of diagnostically used antibodies are not uncommonly found if an extensive normal tissue screening is executed. For example, we had recently observed non-specific staining of smooth-muscle for the PLAP antibody clone 8A9^27^, spermatocytes of the testis for the DOG1 clone SP31^28^, and of corpus luteum of the ovary, adrenal cortical cells, decidua cells for the SATB2 clone 384R-18^29^.

Antibody cross-reactivity does not necessarily represent a significant limitation to the utility of an antibody and can even be considered advantageous. Cross-reactive binding of Melan A clone A103 to adrenocortical cells is for example used as a diagnostic feature for distinguishing adrenocortical tissue from clear cell renal cell carcinoma^30^. The thorough analysis of >4582 tumors from 90 different tumor types demonstrated in this study, that the cross-reactivities detected for our two CTLA-4 antibodies hindered the quantitation of CTLA-4^+^ lymphocytes in only few tumor entities. Because the artefact prone tumor entities were antibody-specific and did not overlap for our antibodies, the use of just two antibodies enabled a successful analysis of the entire tumor set although a few individual tumors such as heavily pigmented melanoma cases remained uninformative for both antibodies. It is of note, that several earlier IHC studies had described CTLA-4 to occur in tumor cells of malignant melanoma^31^, breast cancer^32^, and esophageal carcinomas^33^. Given the complete lack of confirmed tumor cell staining in the 4582 cancers of our study, it appears possible that these earlier reports were based on non-specific antibody binding to tumor cells.

The fact that the analysis of more than 4000 tumor samples from 90 different tumor entities was executed using the same deep-learning algorithm for both antibodies was a major strongpoint of this study and enabled a fully reproduceable evaluation of non-specific staining for multiple antibodies. Thus, the Artificial Intelligence (AI) framework for the detection of non-specific staining reaction was trained on immunostaining of both antibodies—in an equal proportion—to ensure a good performance for both antibody clones. To cover such a wide range of different staining patterns of multiple antibodies across various tumor entities, the AI framework was based on an AI for cell segmentation and the pivotal AI for detecting non-specific antibody staining. However, a major hurdle in developing an AI specific for non-specific staining was to achieve a great diversity of non-specific staining patterns as well as specific lymphocytic staining patterns in the training set. Here, we took advantage of the fact that in most tumor entities the staining quality of both antibodies was complementary to each other (i.e., at least one of the antibody clones showed a specific immunostaining), which dramatically increased the accuracy of our AI. In addition, another advantage of CTLA-4 was the fundamental differences in the shape of CTLA-4^+^ lymphocytes and non-specific staining. Therefore, the AI approach described in this study can be particularly effective in case of lymphocyte markers. For the future, the purpose of this AI approach is—similar to other AI based decision support systems in pathology^34^—to assist the pathologist by excluding >90% of unimportant tumor samples and pointing out the TMA cores of interest (i.e., with potential non-specific staining). Taken together, integrating an AI framework in the process of antibody validation might result in an efficient semi-automated workflow for quality assessment of new antibody clones.

Several data generated from our tumor cohort suggest a possible biological relevance of CTLA-4^+^ lymphocytes. Although the prognostic role of CTLA-4 has been reported contradictory^35^, the fact that the density of CTLA-4^+^ lymphocytes varied between tumor types as well as between individual tumors and that the CTLA-4 density was lower in tumors of advanced clinicopathological parameters was expected because similar findings had been observed for an inflamed immune phenotype^36–38^, CD3^+^^39^, CD8^+^^36^, and CD4^+^^40^, lymphocytes as well as for PD-L1^+^ immune cells^41^ or CD112R^+^ lymphocyte subsets^21^. For the same reason, the significant link between a high number of CTLA-4^+^ cells and PD-L1 expression in tumor cells or tumor associated inflammatory cells is also consistent with the literature^42^. Despite the expected general link between high absolute numbers of CTLA-4^+^ cells and favorable tumor features, there were also some associations between a high CTLA-4/CD3-ratio and favorable tumor features. The latter finding would clearly fit with the concept that immune checkpoint receptors—such as CTLA-4—are upregulated in T-cell accumulations in the tumor micro-environment, so that a high immune checkpoint expression functions as a surrogate for a high number of T-cell accumulations (i.e., a high T-cell density, an inflamed immune phenotype)^21,43–45^. Given that the CD3 density was quantified in an earlier study on non-consecutive slides, it is possible that some associations with clinicopathological parameters might be underrated in this study. Several other studies have also suggested that a high expression of CTLA-4^+^ on T-cells is linked to a favorable disease outcome or tumor features in 289 squamous cell lung cancer^46^, 162 testicular germ cell tumors^47^, 130 breast cancers^32^, 45 mesothelioma patients^48^, and 39 B-cell chronic lymphocytic leukemia^49^.

In summary, CTLA-4^+^ cells could be rapidly and precisely quantitated in this study despite inherent limitations of available CTLA-4 antibodies. The use of two independent antibodies enabled our AI to automatically distinguish “true” from “false” immunostaining and enabled the identification of potentially relevant biologically data such as a link between a low ratio of CTLA-4/CD3 and pN as well as PD-L1^+^ immune cells. Further investigations on the role of CTLA-4^+^ lymphocyte subsets by multiplex fluorescence IHC will most likely benefit from using similar approaches as described here.

## Supplementary information

Supplementary Material

## Data Availability

The datasets used and/or analyzed during the current study are available from the corresponding author on reasonable request.
