# Supplementary material for: Semi-automated validation and quantification of CTLA-4 in 90 different tumor entities using multiple antibodies and artificial intelligence
Source: Lab Invest. 2022 Jan 29;102(6):650–7. doi: 10.1038/s41374-022-00728-4 (PMC9162915; doi:10.1038/s41374-022-00728-4)
Supplement: Supplementary file 1 — Supplementary Material [file 41374_2022_728_MOESM1_ESM.pdf]

Supplementary Appendix

Manuscript title: Semi-automated validation and quantification of CTLA-4 in 90 different Tumor entities using multiple antibodies and artificial intelligence.

Section S1: Supplementary Figures..1

Figure S1: CTLA-4 antibody comparison. 1

Figure S2: Deep learning network for detecting aberrant antibody staining.........................2

Figure S3: Fraction of non-specific staining for each patient. 3

Figure S4: Pearson's correlation of the CTLA-4 density (cells/mm^2) for both antibody clones. 4

Figure S5: Pearson's correlation between the CD3 and CTLA-4 density (cells/mm^2). 4

Section S2: Supplementary Tables..5

Table S1: CTLA-4+ cell densities (cells/mm2) in 90 different tumor types. 5

Section S1: Supplementary Figures

Figure S1: CTLA-4 antibody comparison.

(A-B) Multiplex fluorescence IHC of the used CTLA-4 antibody clone MSVA-152R (green) and CAL49 (red) revealed a high degree of co-expression (orange). 100x magnifications are shown in A and 400x magnifications are shown in B. (C) The CTLA-4 expression level and CTLA-4^{+} cell density was highly concordant across 35 representative areas in human tonsil.

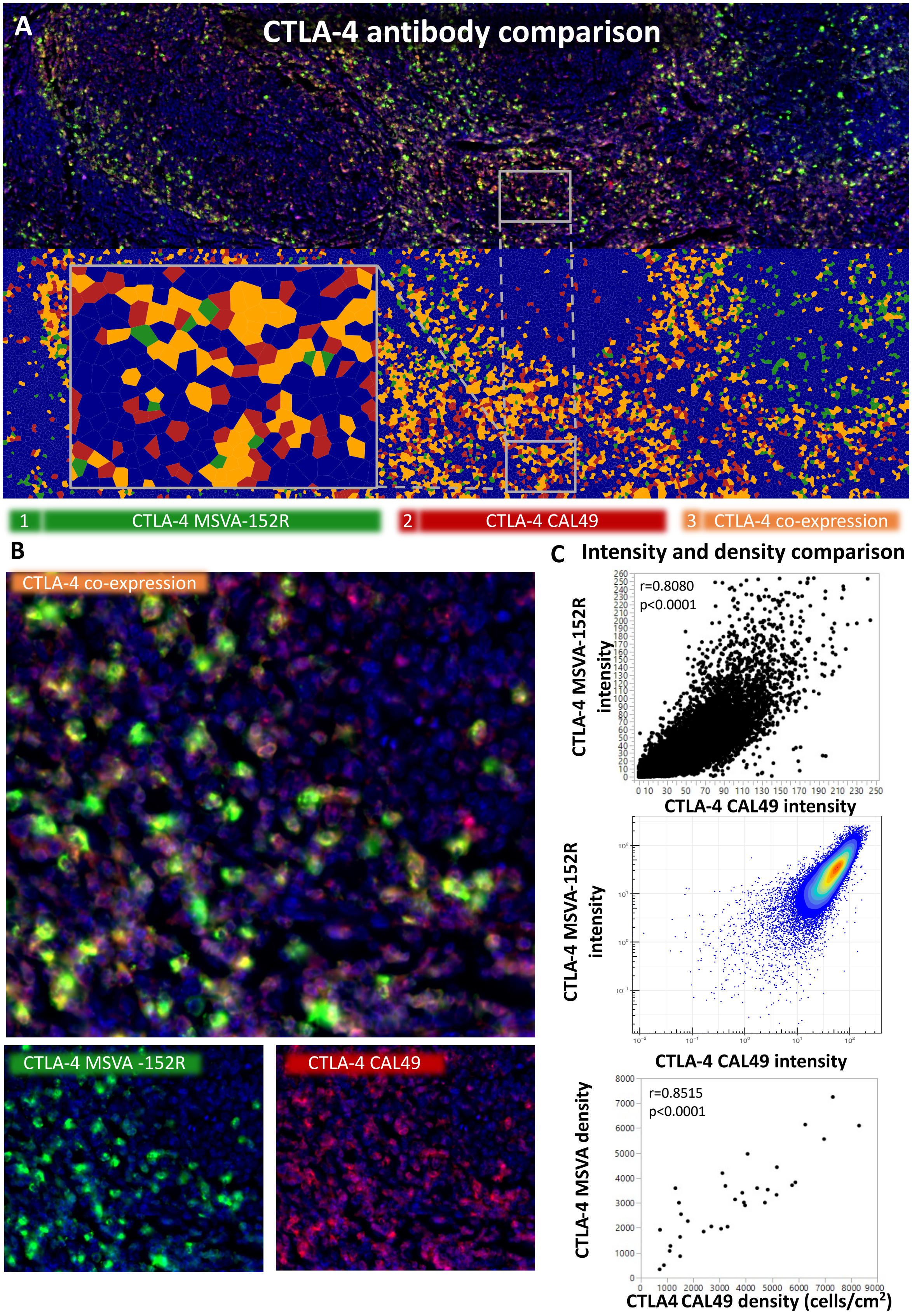

Figure S2: Deep learning network for detecting aberrant antibody staining.
(A)Tissue microarrays were stained for the comparison of two different CTLA-4 antibody clones (MSVA-152R and CAL49). (B)A pretrained convolutional neural network (U-Net) for cell (white) identification (black indicates background) was used and showed a high performance in area under receiver operating characteristic curves (AUC). (C) A DeepLab3[+] was trained to identify aberrant antibody staining (false positive, white). (D) Both deep learning systems were combined to identify false positive antibody staining and to compensate for such antibody shortcomings. (E) Thus, the deep learning-based framework facilitates studies on biological relevant targets using antibodies with false positive staining in some tumor entities.

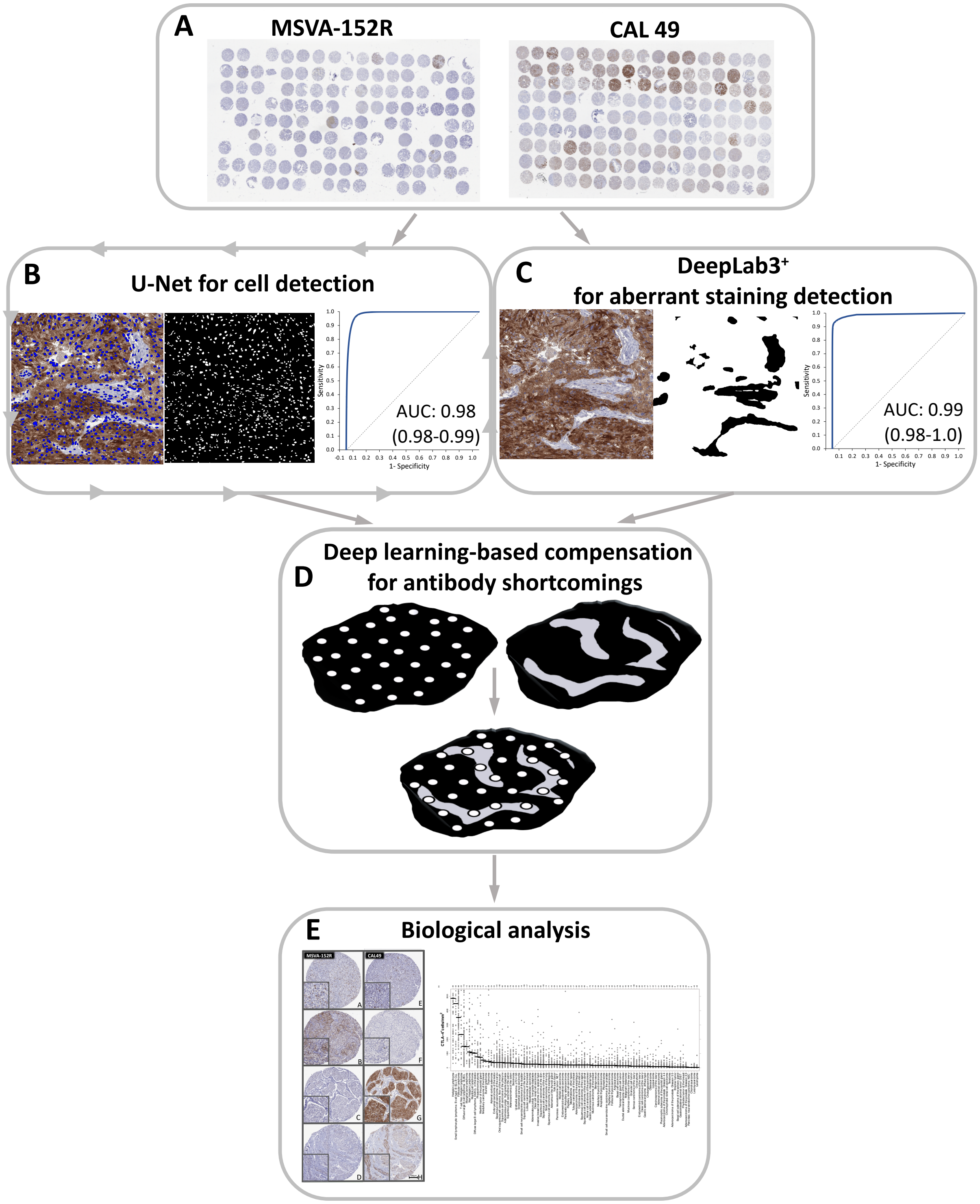

Figure S3: Fraction of non-specific staining for each patient.
The fraction of non-specific staining for the MSVA-152R (A) and CAL49 (B) clone is depicted for every individual patient. The black line indicates the 5% threshold that was used to identify patients with a driving component of aberrant antibody staining.

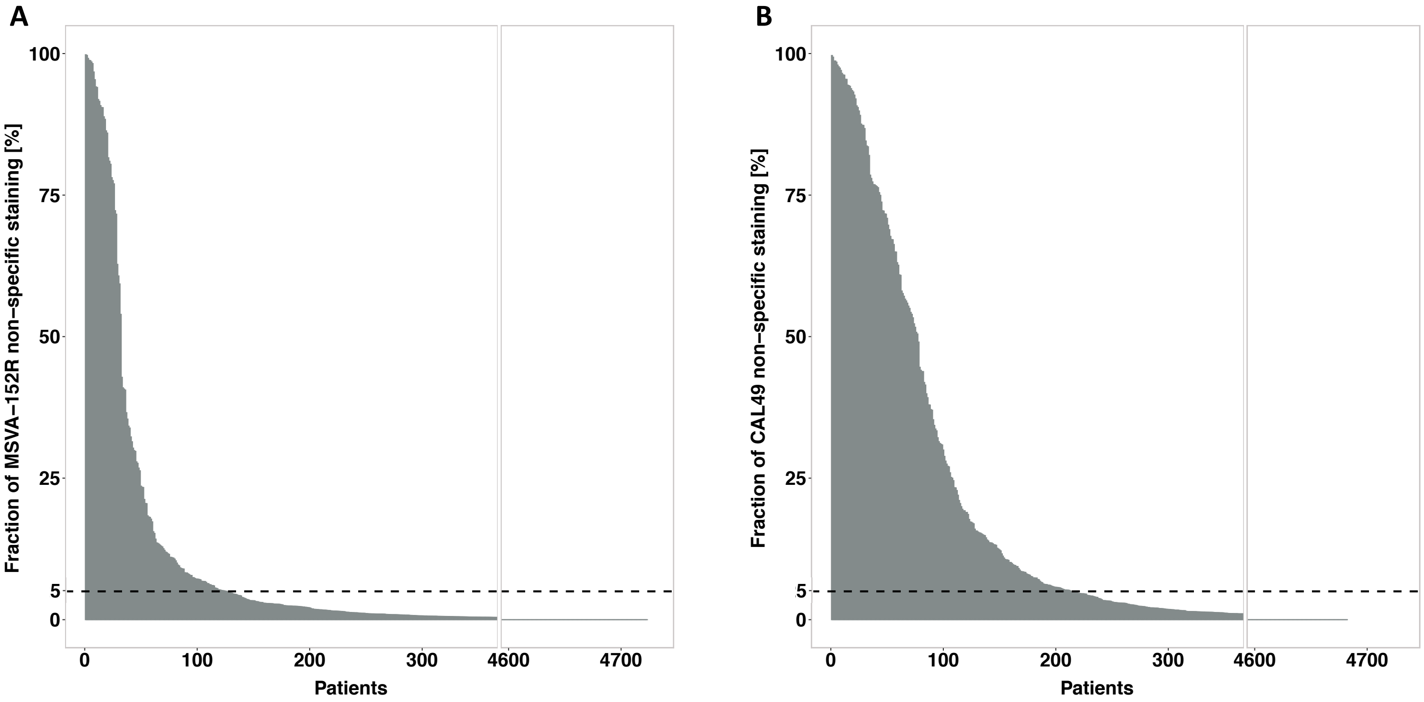

Figure S4: Pearson's correlation of the CTLA-4 density (cells/mm²) for both antibody clones.

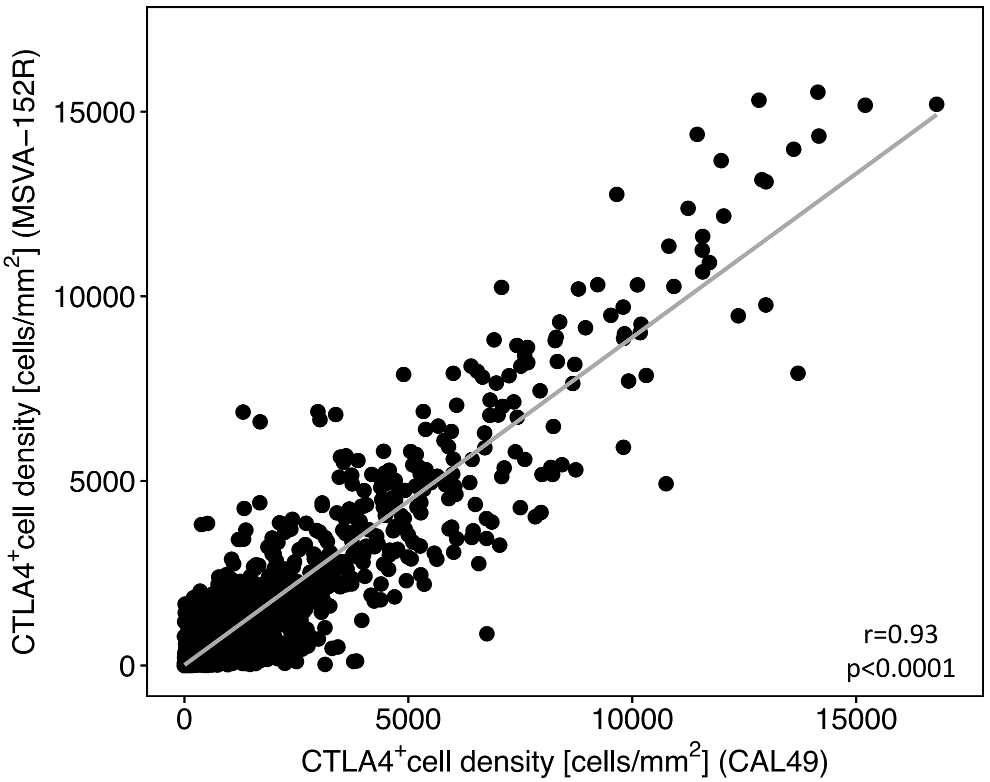

Figure S5: Pearson's correlation between the CD3 and CTLA-4 density (cells/mm²).

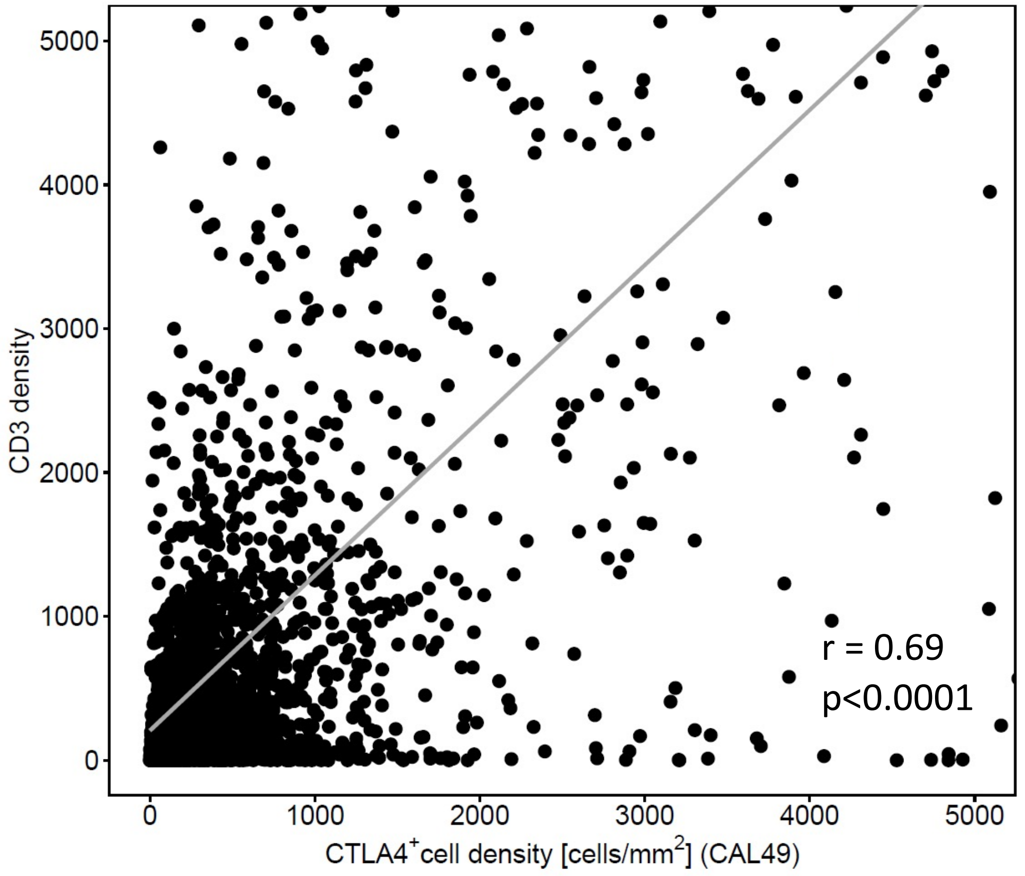

Section S2: Supplementary Tables

Table S1: CTLA-4+ cell densities (cells/mm2) in 90 different tumor types.

Statistic	n	Mean	St. Dev.	Min	Pctl(25)	Pctl(75)	Max
Hodgkin Lymphoma	96	5,916	3,826	313	2,588	8,894	14,909
Small lymphocytic lymphoma, B-cell type (B-SLL/B-CLL)	50	5,357	4,128	325	1,973	6,755	15,999
Follicular lymphoma	88	3,890	2,063	525	2,331	4,935	11,318
T-cell Non Hodgkin lymphoma	23	2,701	1,949	225	1,003	3,812	6,563
Diffuse large B cell lymphoma (DLBCL)	114	2,048	1,836	91	549	2,724	7,356
Marginal zone lymphoma	15	1,510	919	305	756	1,816	3,158
Non-Hodgkin Lymphoma	60	2,080	2,271	33	515	2,885	12,830
Mantle cell lymphoma	18	1,353	1,003	339	763	1,652	4,444
Diffuse large B-cell lymphoma (DLBCL) in the testis	16	1,343	1,005	213	567	1,896	3,690
Phaeochromocytoma	48	1,084	1,275	8	295	1,254	6,139
Warthin tumor of the parotid gland	47	1,274	1,409	3	354	1,674	6,107
Medullary carcinoma of the breast	15	767	716	77	391	850	2,697
Burkitt lymphoma	4	528	415	103	323	663	1,092
Thymoma	28	1,479	2,162	26	119	1,471	8,811
Adrenal cortical adenoma	24	473	313	159	228	554	1,198
Embryonal carcinoma of the testis	24	529	535	49	164	672	2,394
Squamous cell carcinoma of the cervix	121	522	600	25	146	673	3,807
Oral squamous cell carcinoma (floor of the mouth)	130	508	503	9	182	704	3,299
Squamous cell carcinoma of the pharynx	60	538	511	4	165	710	2,705
Adenomatous polyp, high-grade dysplasia	48	382	227	31	247	534	1,116
Squamous cell carcinoma of the penis	80	468	523	12	106	608	2,854
Adenocarcinoma of the colon	78	448	343	58	222	602	2,173
Seminoma	36	554	522	26	180	908	2,080
Urothelial carcinoma, pT2-4 G3	33	419	347	35	150	588	1,292
Endometrial serous carcinoma	34	404	406	26	125	514	1,925
Small cell neuroendocrine carcinoma of the bladder	18	579	812	7	110	742	3,316
Squamous cell carcinoma of the larynx	105	376	431	11	116	461	3,189
Lobular carcinoma of the breast	117	345	308	30	129	459	1,506
Hepatocellular carcinoma	47	344	339	6	138	377	1,407
Adenomatous polyp, low-grade dysplasia	45	444	563	92	168	451	3,095
Clear cell renal cell carcinoma	45	316	277	13	121	361	1,471
Invasive breast carcinoma of no special type	80	489	687	45	139	544	4,077
Squamous cell carcinoma of the skin	82	355	391	21	98	430	2,318
Squamous cell carcinoma of the vulva	123	378	412	8	112	486	2,476

Small cell carcinoma of the lung	13	431	402	34	150	831	1,262
Squamous cell carcinoma of the esophagus	71	338	306	20	101	477	1,340
Adenoma of the thyroid gland	43	396	537	17	115	436	2,907
Yolk sack tumor	29	407	469	33	144	365	1,966
Pancreas, neuroendocrine tumor (NET)	41	367	438	22	120	414	1,982
Merkel cell carcinoma	38	369	399	10	123	471	1,427
Endometrioid endometrial carcinoma	47	268	249	14	78	333	897
Pancreatic/Ampullary adenocarcinoma	28	433	979	45	122	358	5,348
Papillary renal cell carcinoma	32	280	307	18	112	383	1,641
Mesothelioma, other types	34	281	319	25	110	316	1,622
Tubular carcinoma of the breast	17	325	316	21	116	444	1,230
Adenocarcinoma of the esophagus	82	303	318	32	123	350	1,945
Carcinosarcoma of the uterus	20	482	937	11	61	306	3,875
Squamous cell carcinoma of the vagina	53	365	474	21	111	385	2,518
Squamous cell carcinoma of the anal canal	83	304	320	9	111	382	1,825
Acinar cell carcinoma of the pancreas	7	284	306	33	141	269	951
Gastric adenocarcinoma, intestinal type	79	295	348	23	105	347	2,489
Mucinous carcinoma of the breast	16	264	352	20	90	224	1,473
Benign nevus	24	283	208	45	135	426	783
Medullary thyroid carcinoma	36	231	217	20	88	270	941
Anaplastic thyroid carcinoma	23	270	325	45	106	282	1,589
Pilomatrixoma	31	213	190	14	71	289	811
Small cell neuroendocrine carcinoma of the prostate	14	276	317	11	77	314	1,182
Papillary thyroid carcinoma	43	257	267	4	87	332	1,371
Follicular thyroid carcinoma	46	341	574	10	86	283	3,383
Angiosarcoma	27	349	538	60	114	310	2,736
Basal cell carcinoma	78	285	302	5	84	400	1,397
Tenosynovial giant cell tumor	38	243	333	23	74	227	1,643
Ductal adenocarcinoma of the pancreas	81	189	184	25	90	210	1,226
Malignant melanoma	40	321	400	17	76	376	1,537
Mucinous carcinoma of the ovary	48	195	214	18	67	219	1,018
Granular cell tumor	18	255	357	56	92	233	1,557
Serous carcinoma of the ovary	128	240	361	6	66	297	3,186
Oncocytoma	36	217	242	17	57	289	1,028
Endometrioid carcinoma of the ovary	66	271	393	12	58	242	2,164
Clear cell carcinoma of the ovary	22	188	183	20	46	304	656
Gastric adenocarcinoma, diffuse type	79	148	156	6	55	170	811
Cholangiocarcinoma	47	181	247	6	51	206	1,357
Osteosarcoma	17	178	232	20	45	181	948
Liposarcoma	42	163	220	6	36	153	956

Carcinosarcoma of the ovary	17	268	344	11	66	310	1,254
Adrenal cortical carcinoma	21	222	324	9	35	289	1,475
Pleomorphic adenoma of the parotid gland	44	205	377	5	33	199	1,908
Adenocarcinoma of the prostate, Gleason 4+4	80	127	150	20	45	130	837
Chromophobe renal cell carcinoma	36	160	194	9	26	228	702
Teratoma	38	171	235	5	43	188	1,078
Adenocarcinoma of the prostate, Gleason 5+5	85	114	133	7	41	137	786
Brenner tumor	8	168	225	23	26	200	669
Basal cell adenoma of the salivary gland	15	283	685	9	31	143	2,711
Gastrointestinal stromal tumor (GIST)	49	164	244	0	21	181	1,167
Phyllodes tumor of the breast	49	139	271	0	20	136	1,588
Adenocarcinoma of the prostate, Gleason 3+3	83	89	74	8	32	121	386
Pancreas, neuroendocrine carcinoma (NEC)	2	40	15	29	35	45	51
Chondrosarcoma	8	82	96	9	25	99	249
Leiomyosarcoma	43	62	77	0	14	64	316
Leiomyoma	49	71	175	0	6	52	880
